# Pulse Width Modulation Electro-Acupuncture on Cardiovascular Remodeling and Plasma Nitric Oxide in Spontaneously Hypertensive Rats

**DOI:** 10.1093/ecam/neq063

**Published:** 2011-03-13

**Authors:** Xuan Xiong, Chao You, Qiu-Chao Feng, Ting Yin, Zhong-Ben Chen, Patrick Ball, Le-Xin Wang

**Affiliations:** ^1^Department of Biomedical Engineering, Zhongshan School of Medicine, Sun Yat-Sen University, Guangzhou, Guangdong, China; ^2^Guangzhou Red Cross Hospital, Guangzhou, Guangdong, China; ^3^School of Biomedical Sciences, Charles Sturt University, Wagga Wagga, NSW 2678, Australia

## Abstract

This study was designed to investigate the effect of pulse width modulation electro-acupuncture (PWM-EA) on cardiovascular remodeling and nitric oxide (NO) in spontaneously hypertensive rats (SHR). Thirty-four male SHR were randomly divided into control, captopril, and two PWM-EA groups, which were treated with 350 Hz (SHR-350 Hz) and whole audio bandwith electro-acupuncture (SHR-WAB group) respectively, on the ST 36 point located on the outside of the hind leg. Systolic blood pressure (BP), plasma and myocardial NO were measured. Histological studies were also performed on the aortic wall and the left ventricle. The BP in the SHR-350 Hz, SHR-WAB and the captopril groups was lower than in the control group following the treatment (*P* < .05). The average aortic media wall thickness in the two electro-acupuncture groups was less than in the control group (*P* < .05). The left ventricle/heart weight ratio in the captopril and SHR-350 Hz groups was less than in the control group (*P* < .01), but was similar between the SHR-WAB and the control group (*P* > .05). The plasma and myocardium NO levels were elevated in the captopril and the SHR-350 Hz group (*P* < .05 and .01, resp.). The plasma level of NO in the SHR-WAB group was also higher than in the control group (*P* < .05). We concluded that pulse width modulation electro-acupuncture on the ST 36 point prevents the progression of hypertension and diminishes the cardiovascular remodeling in SHR. It also elevates plasma and cardiac NO in this animal model.

## 1. Introduction

Left ventricular (LV) hypertrophy and vascular remodeling are the two common pathological findings in patients with hypertension. Prevention and reversal of LV hypertrophy and vascular remodeling are some of the major therapeutic objectives for hypertension management. Non-pharmacological treatment of hypertension is an important part of the therapeutic strategy. Acupuncture is one such strategy, although some have claimed that the supporting evidence is equivocal [1, 2]. However, well conducted studies have shown benefit [3, 4] and it has been suggested that methodological issues may be responsible for some of the ambiguity [5]. Previous work has indicated that music, acupuncture and electro-acupuncture can reduce blood pressure (BP) and contribute to the overall management of hypertension [4, 6, 7]. There is also evidence showing that acupuncture or electro-acupuncture leads to improvements in endothelial cell function, which may help with BP control [8–10].

A major drawback with the conventional pulse electro-acupuncture for BP control is the attenuation of the therapeutic effect over time [11, 12]. Musically pulsed electro-acupuncture uses music as its output waveform signal, or the music signal is superimposed on the periodic pulse. With these devices, the amplitude of the output pulse varies music amplitude, which is undesirable in the delivery of ongoing therapy. We have developed a pulse width modulation electro-acupuncture (PWM-EA) device for hypertension treatment. The main feature of this unit is to regulate the pulse duty factor of output pulses (the ratio between the pulse duration and pulse interval), according to the amplitude variation of the audio signals. The theoretical advantage of this new device is that it avoids attenuation of therapeutic effect in the conventional acupuncture devices, and provides stimulation at constant pulse amplitude.

Our pilot study showed that electro-acupuncture with a pulse width modulation at 350 Hz or at the whole audio bandwidth (WAB) reduces the BP in hypertensive rats [13]. However, the effect of PWM-EA on the cardiovascular remodeling secondary to hypertension is uncertain. The primary purpose of this study was to investigate if the new PWM-EA would be able to diminish the ventricular hypertrophy or vascular wall thickening in a hypertension rat model. In addition, the effect of PWM-EA on the plasma and cardiac levels of nitric oxide (NO) was also evaluated.

## 2. Methods

This study was approved by the Institutional Review Board of the Sun Yat-Sen University. All procedures were in accordance with institutional guidelines for animal research. Thirty-four male spontaneous hypertension rats (SHR, 13 weeks) were provided by Vital River Company (Beijing, China). They were maintained in a quiet room at constant temperature (20–22°C) and humidity (50–60%) with 12-h light–dark cycle. Systolic BP was measured in all animals by a standard tail-cuff method with sphygmomanometer. Captopril was obtained from Shanghai Squibb Pharmaceuticals. Signal processing and observation was performed with V989 VCD (Guangdong Diwa Electronic Technology, China) and TDS2012 Digital Oscilloscope (Tektronix Inc, USA). We used our own PWM-EA to treat SHR.

### 2.1. Animals and Treatment

The SHRs were randomly divided into captopril (administered by gastric gavages, 15.625 mg kg^−1^ per day), the SHR-350 Hz (electro-acupuncture with pulse width modulated by 350 Hz), PWM-EA (electro-acupuncture with pulse width modulated by whole audio-frequency), and control groups.

All treatments were administered in the mornings, once daily for the first five days in a week. The SHR-350 Hz and PWM-EA groups received electro-acupuncture treatment for 30 min per day. The acupuncture of both groups was conducted in a ST 36 point (“Zusanli"), which is located on the outside of the hind leg, just below the knee, and outside of the tibial crest. An adhesive silver electrode patch was attached to each side of the ST 36 points for the delivery of the stimulation. The intensity of the electrical stimulation was adjusted between 2–6 V, to the point where there was no noticeable muscle stimulation. After 8 weeks of treatment, rats were euthanized with sodium pentobarbital (2%, 1 ml, IP) followed by removal of the heart and aorta for pathological examination.

### 2.2. Measurement of Blood Pressure

Systolic BP was measured before the treatment and in the third, sixth, and eighth week during the treatment in conscious SHR by an indirect tail-cuff method. The measurement was performed in the same period of the day (morning). Rats were preheated at 30°C for 10 min, and then three consecutive measurements of BP were averaged.

### 2.3. Determination of NO in Myocardium and Plasma

Blood was collected from the right ventricle before the heart was arrested. Two milliliters of blood was placed into a tube, centrifuged 3000 rpm for 10 min. The supernatant plasma was stored at –80°C. After weighing the heart, 200 mg myocardial tissue was homogenized with 1.8 ml physiological saline (0.86%). The homogenization was performed on ice-bath (4°C) and the homogenates were centrifuged at 4°C, 3000 rpm for 10 min. The supernatant was stored at –80°C. The NO concentration was assayed with the Greiss reagent (Nanjing Jiancheng Bioengineering Institute, China), after reduction with nitrate reductase. The resulting supernatant of sample mixture with medium was measured at 550 nm on a spectrophotometer (Pgeneral, T60 UV-VIS, China). Conditioned medium from untreated samples with no added Greiss reagent were used as sample blanks. Known concentrations of KNO_2_ and KNO_3_ were used as standards. All procedures were carried out according to the manufacturer's guide.

### 2.4. Tissue Preparation and Histopathology

Part of the left ventricle and thoracic aorta were segregated, fixed in 10% formaldehyde and embedded in paraffin. The tissues were sectioned in 5 *μ*m, and stained with hematoxylin-eosin (HE). The hearts were rinsed for 1 min in 0.01 mol/l cold PBS (pH 7.4). The weight of the heart and the left ventricle were determined by electronic balance (Mettler-Toledo International Inc, Switzerland). The heart/body and left ventricle/heart weight ratios were calculated.

The pictures were collected by an LC evolution digital camera of an Olympus IX-71 microscope. For the sections of the aorta, maximal and minimal internal and external diameters were determined at 10x magnification. These values were used to determine the mean values of internal and external diameters. Wall thickness was calculated by subtraction of the two diameters. Image Prolus software (version 5.01, Media Cybemetics, Silver Spring, USA) was used to determine cross-sectional areas of the different aortic layers including the media layer. Finally, the media thickness of the aorta was calculated as the distance from the external elastic lamina (EEL) to the internal elastic lamina (IEL). The cross-sectional area of the media layer is defined as the result of the EEL area minus IEL area.

### 2.5. Statistical Analysis

All data are presented as the mean ± SD. Two-way ANOVA followed by a post hoc multiple comparison (Duncan's test) was used to analyze the differences among treatments, across times, as well as between their interactions. Categorical data were analyzed by Chi-square test. The relationship between post-treatment BP and the parameters for LV or aortic remodeling was analyzed with Pearson's. *P* < .05 was considered statistically significant.

## 3. Results

### 3.1. Systolic Blood Pressure

**Figure 1:**
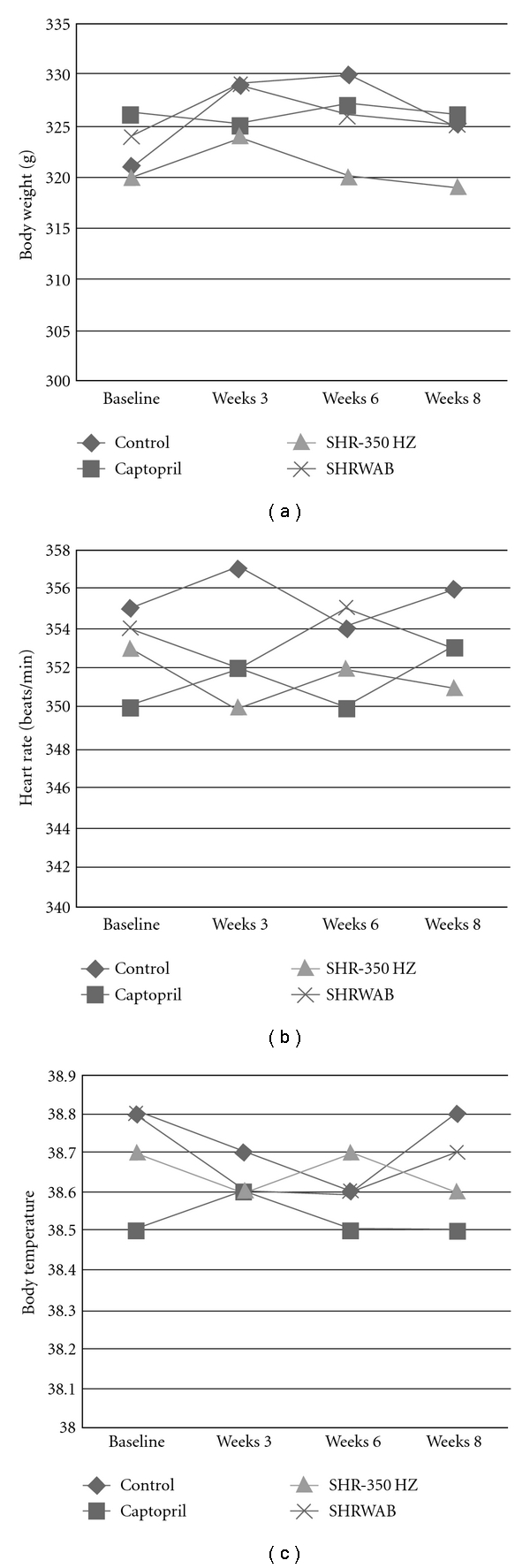
Mean values of body weight (a), heart rate (b) and body temperature (c) before and after the experiment. There was no significant difference between the four time points in the four groups (*P* > .05). The baseline body weight, heart rate and body temperature were similar between the four groups (*P* > .05).

Body weight, heart rate and body temperature of the four groups remained unchanged at the end of the study (*P* > .05, Figure 1).  Table 1 shows the base line and post-treatment systolic BP. There was no significant difference in the baseline systolic BP between the control and the treatment groups (*P* > .05). The average baseline systolic BP in the SHR-WAB and the captopril group was slightly higher than in the SHR-350 Hz group but this did not reach statistical significance (*P* > .05). In the control group, BP gradually elevated during the 8-week study period (*P* < .01). There was a significant reduction in systolic BP in the captopril group following the treatment (*P* < .01). There was no significant difference in the mean systolic BP between Week 3, 6 and 8 in this group (*P* > .05). In the SHR-350 Hz group, the mean systolic BP at Week 3 was lower than the baseline value (*P* < .05), but the mean systolic BP in Week 6 and 8 was similar to the baseline (*P* > .05) and higher than in Week 3 (*P* < .05). In the SHR-WAB group, there was no significant reduction in BP at the end of the 8-week treatment (*P* > .05). In the captopril and the two electro-acupuncture groups, the mean systolic BP at the end of treatment was lower than in the control group (*P* < .01 and *P* < .05, resp.). 

### 3.2. Left Ventricular Hypertrophy

Cardiac hypertrophy was estimated on the basis of the left ventricle/heart ratio and the heart/body ratio. As shown in Table 2, there was no significant difference in the heart/body ratio, or the left ventricle/heart ratio among the four groups before the treatment (*P* > .05). In the captopril group, there was a significant decrease in the heart/body ratio and left ventricle/heart ratio following the treatment (*P* < .05). There was also a significant reduction in the left ventricle/heart ratio in the SHR-350 Hz group (*P* < .05). The left ventricle/heart ratio in the SHR-WAB group remained unchanged at the end of the experiment (*P* > .05). There was no significant correlation between the Week-8 systolic BP and the left ventricle/heart or heart/body ratio in the four groups (*P* > .05). 

### 3.3. Thickness of Aorta

Morphological data obtained from sections of the aorta are shown in Table 3. Compared with the control group, the media thickness of the aorta, the media-to-lumen diameter ratio, and the media area were significantly lower in the three treatment groups (*P* < .05 and .01, resp.). The media thickness, media-to-lumen diameter ratio, and the area of the media in the captopril group were similar to those in the SHR-350 Hz group (*P* > .05). There was no significant correlation between the Week-8 BP and media thickness, the media-to-lumen diameter ratio, or the media area in the four groups (*P* > .05). 

### 3.4. NO

The baseline and post-treatment plasma and myocardial NO are shown in Table 4. There was no significant difference in the plasma or myocardial NO between the four groups before the treatment (*P* > .05). Following the treatment, the plasma NO levels were elevated in the captopril and the two acupuncture groups (*P* < .01 resp.). The mean plasma NO in the three treatment groups was higher than in the control group (*P* < .05 and .01, resp.). The myocardial NO in the captopril and the SHR-350 Hz group was also elevated following the treatment (*P* < .05), but the increase in the myocardial NO in the SHR-WAB group did not reach statistical significance (*P* > .05). The post-treatment myocardial NO in the captopril and the SHR-350 Hz group was higher than in the control group (*P* < .05). 

## 4. Discussion

The main findings of our study are: (i) Pulse width modulation electro-acupuncture prevented the progressive increase in systolic BP in SHR; (ii) The electro-acupuncture was also associated with a reduction in the thickness of the aortic wall, and 350 Hz electro-acupuncture was associated with a reduction in left ventricle/heart ratio; (iii) The plasma and myocardium NO levels were elevated following the electro-acupuncture treatment.

The meridian theory, which deals with physiologic regulation and pathologic changes in the human body, is an important component of traditional Chinese medicine. The theory provides an essential guide for diagnosis and treatment strategies in oriental medicine, in which acupuncture remains as a central part [14, 15].

The stomach meridian begins from the lateral side of the nose and ascending laterally along the infraorbital ridge [16]. After passing through the diaphragm, it enters the stomach and connects with the spleen and the heart. Subsequently, it descends inside the abdomen and reaches the knees. Eventually, it ends at the lateral side of the tip of the second toe [16]. Based on this meridian theory, we postulate that acupuncture on the stomach meridian would treat disorders originated from stomach, spleen or the heart. “Zusanli" or ST 36 has been used in several animal models to investigate the effect of acupuncture on gastrointestinal and cardiovascular systems [17, 18]. Acupuncture on ST 36 is also known to reduce BP [9, 19, 20], but there is a paucity of data for studying the effect of ST 36 stimulation on the remodeling of the heart and the level of NO in the cardiac tissues.

In our study, captopril, a first generation angiotensin-converting enzyme inhibitor, decreased BP in the SHR. Following the pulse width modulation electro-acupuncture, there was no significant reduction in the BP. However, BP in the electro-acupuncture groups remained unchanged after 8 weeks of the therapy, while the BP in the control group was significantly elevated over the same period. These results indicate that the pulse width modulation electro-acupuncture may influence the progression of high BP in this animal model.

Previous studies have indicated that acupuncture of the ST 36 points elevates the levels of NO in the central nervous system or the peripheral blood [8, 9, 20]. In our study, electro-acupuncture with 350 Hz pulse width modulation or modulation by whole audio-frequency was associated with a significant increase in plasma NO. Electro-acupuncture with 350 Hz pulse width modulation was also associated with an increase in the NO levels in the cardiac tissues. These results indicate that the effect of the pulse with modulation electro-acupuncture on BP may be related to an enhanced biosynthesis or release of NO.

**Figure 2:**
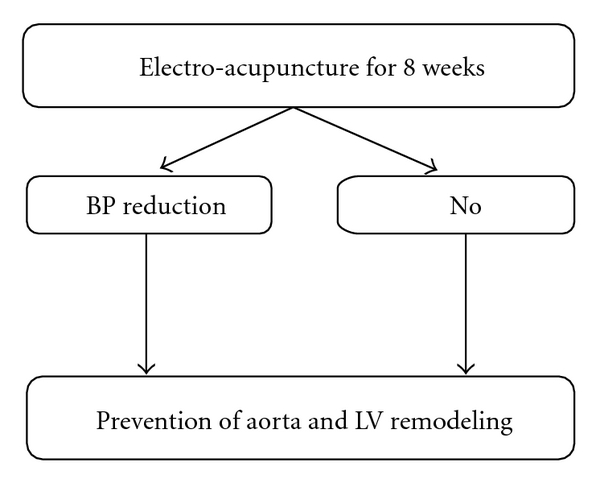
Potential mechanisms by which electro-acupuncture diminished aortic and LV remodeling following 8-week treatment. Both hypertension control and enhanced biosynthesis of NO may be involved in the prevention of aortic and LV remodeling.

LV hypertrophy and aortic thickening are two common pathological findings in hypertensive animals [21]. In the present study, similar to captopril treatment, 350 Hz pulse with modulation electro-acupuncture was associated with a lower left ventricle/heart ratio. The media area and the media-lumen diameter ratio of the aorta in the two electro-acupuncture groups were smaller than in the control group. These results suggest that pulse width modulation electro-acupuncture diminishes the remodeling of the heart and the aorta in SHR. The signaling pathways by which the pulse width modulation electro-acupuncture influences cardiovascular remodeling are unclear. Although in the SHR-350 Hz group there was a noticeable reduction in BP at Week 3 of the treatment, the mean systolic BP at the end of the experiment was similar to the baseline. There was no significant correlation between the Week-8 BP and the left ventricle/heart ratio or the parameters for aortic remodeling. BPs in the SHR-WAB group remained unchanged, and no correlation between the Week-8 BP and remodeling parameters was indentified. Given that hypertension is clearly responsible for the heart and aortic remodeling in SHR [21], and we only included eight animals in each group in the present study, a future study in a larger number of animals may be required to demonstrate the impact of hypertension control on cardiovascular remodeling (Figure 2). The increase in plasma and myocardial NO following the electro-acupuncture treatment may also be related to the remodeling attenuation, but this is yet to be confirmed by further investigations in a larger number of animals (Figure 2). 

A potential clinical application of the present study is that the pulse width modulation electro-acupuncture on ST 36 point may help to manage human hypertension. The technique is noninvasive and each treatment session may take 30 min to complete. Clinical trials are required to ascertain if this treatment alone could reduce BP and prevent cardiovascular remodeling in patients with hypertension.

One of the limitations of the present study is that a relatively low voltage of electrical stimulation (2–6 V) was used for the electro-acupuncture, because only low-intensity electro-acupuncture produces an antihypertensive effect [22]. Musically pulsed electro-acupuncture techniques were initially developed for human models. The application of these techniques has not been subject to robust and systematic validation. We also used conductive skin patches, instead of conventional needles, for the delivery of the electrical stimulation. This is largely because upholding a needle over a long period time during the acupuncture is technically challenging. The therapeutic effect of needle acupuncture and acupuncture delivered through skin patches has not been fully studied, but in human subjects, electrical stimulation via skin patch electrodes is as effective as needle electro-acupuncture in pain relief [23]. It is unclear if skin patch electrodes and needle electrodes offer the same therapeutic effects in hypertension animal models. Finally, we did not perform follow up studies, thus, the medium- to long-term antihypertensive effects of the electro-acupuncture on ST 36 points remain to be seen.

In conclusion, pulse width modulation electro-acupuncture on ST 36 point restrains the escalation of hypertension in SHR. This novel treatment also diminishes the cardiac and aortic remodeling in this animal model. Pulse width modulation electro-acupuncture elevates plasma NO, and with 350 Hz modulation, it also stimulates the biosynthesis of NO in the cardiac tissues.

## Figures and Tables

**Table 1:** Effect on the systolic BP.

Groups	*n*	Before	Third week	Sixth week	Eighth week
Control	8	173.8 ± 9.0	184.4 ± 9.3^§^	187.6 ± 8.7^§^	191.0 ± 9.0^§^
		(188.6)	(199.7)	(201.9)	(205.8)
Captopril	9	179.4 ± 8.6	164.0 ± 8.7^∗∗,§^	162.5 ± 8.1^∗∗,§^	166.8 ± 8.3^∗∗,§^
		(193.6)	(178.3)	(175.8)	(180.5)
SHR-350 Hz	9	173.8 ± 8.1	167.3 ± 8.5*	171.6 ± 8.2*	173.5 ± 8.7*
		(187.1)	(181.3)	(185.1)	(187.8)
SHR-WAB	8	178.3 ± 8.6	177.4 ± 8.2	176.3 ± 7.9	174.0 ± 8.6*
		(192.5)	(190.1)	(189.3)	(188.2)

The numbers in the bracket represent the 95th percentile.

*P < .05 versus control; **P < .01 versus control. ^§^ *P* < .01 versus baseline value.

**Table 2:** Effect of treatment on the weight of the left ventricle and the heart.

Groups	*n*	Heart/body ratio (mg g^−1^)		LV/heart ratio (mg mg^−1^)	
		Before	After	Before	After
Control	8	3.61 ± 0.07	2.99 ± 0.09	0.82 ± 0.10	0.80 ± 0.06
Captopril	9	3.19 ± 0.06	2.71 ± 0.04^∗,§^	0.84 ± 0.09	0.74 ± 0.07^∗,§^
SHR-350 Hz	9	2.97 ± 0.09	2.84 ± 0.07	0.86 ± 0.09	0.76 ± 0.12^∗,§^
SHR-WAB	8	3.02 ± 0.06	2.85 ± 0.08	0.81 ± 0.10	0.78 ± 0.14

LV: left ventricle.

*P < .05 versus control group. ^§^ *P* < .05 versus baseline value of the same group.

**Table 3:** Medial thickness of aorta and the cardiomyocyte area.

Group	*n*	MT (*μ*m)	LD (*μ*m)	MT/LD (%)	Media area (mm^2^)
Control	8	119.8 ± 7.2	1602 ± 76.0	7.4 ± 0.6	0.67 ± 0.10
Captopril	9	94.0 ± 5.5**	1709 ± 52.3	5.5 ± 0.4**	0.56 ± 0.06**
SHR-350 Hz	9	102.5 ± 9.1*	1663 ± 74.6	6.2 ± 0.8**	0.61 ± 0.07*
SHR-WAB	8	107.5 ± 8.0*	1657 ± 52.1	6.5 ± 0.7*	0.62 ± 0.08*

MT: media thickness; LD: lumen diameter; MT/LD: media thickness/lumen diameter.

*P < .05 versus control; **P < .01 versus control.

**Table 4:** NO.

Group	*n*	Plasma NO (*μ*mol l^−1^)		Myocardium NO (pmol g^−1^)	
		Before	After	Before	After
Control	8	22.06 ± 4.61	21.58 ± 4.75	1.21 ± 0.20	1.19 ± 0.19
Captopril	9	21.76 ± 5.12	36.07 ± 5.28^∗∗,§^	1.13 ± 0.24	1.83 ± 0.20^∗,Δ^
SHR-350 Hz	9	23.44 ± 5.33	32.65 ± 6.43^∗∗,§^	1.18 ± 0.17	1.68 ± 0.15^∗,Δ^
SHR-WAB	8	21.39 ± 4.86	29.53 ± 5.20^∗,§^	1.26 ± 0.32	1.56 ± 0.14

*P < .05 versus control; **P < .01 versus control. ^§^ *P* < .01 versus baseline of the same group. ^Δ^ *P* < .05 versus baseline of the same group.
